# Low-cost and convenient screening of disease using analysis of physical measurements and recordings

**DOI:** 10.1371/journal.pdig.0000574

**Published:** 2024-09-19

**Authors:** Jay Chandra, Raymond Lin, Devin Kancherla, Sophia Scott, Daniel Sul, Daniela Andrade, Sammer Marzouk, Jay M. Iyer, William Wasswa, Cleva Villanueva, Leo Anthony Celi

**Affiliations:** 1 Harvard Medical School, Harvard University, Boston, Massachusetts, United States of America; 2 Global Alliance for Medical Innovation, Cambridge, Massachusetts, United States of America; 3 Harvard College, Harvard University, Boston, Massachusetts, United States of America; 4 Duke University, Durham, North Carolina, United States of America; 5 Department of Biomedical Sciences and Engineering, Mbarara University of Science and Technology, Mbarara, Uganda; 6 Escuela Superior de Medicina, Instituto Politécnico Nacional, México, D.F., México; 7 Institute for Medical Engineering and Science, Massachusetts Institute of Technology, Cambridge, Massachusetts, United States of America; 8 Division of Pulmonary, Critical Care and Sleep Medicine, Beth Israel Deaconess Medical Center, Boston, Massachusetts, United States of America; 9 Department of Biostatistics, Harvard T.H. Chan School of Public Health, Boston, Massachusetts, United States of America; Brigham and Women’s Hospital, Harvard Medical School, UNITED STATES OF AMERICA

## Abstract

In recent years, there has been substantial work in low-cost medical diagnostics based on the physical manifestations of disease. This is due to advancements in data analysis techniques and classification algorithms and the increased availability of computing power through smart devices. Smartphones and their ability to interface with simple sensors such as inertial measurement units (IMUs), microphones, piezoelectric sensors, etc., or with convenient attachments such as lenses have revolutionized the ability collect medically relevant data easily. Even if the data has relatively low resolution or signal to noise ratio, newer algorithms have made it possible to identify disease with this data. Many low-cost diagnostic tools have been created in medical fields spanning from neurology to dermatology to obstetrics. These tools are particularly useful in low-resource areas where access to expensive diagnostic equipment may not be possible. The ultimate goal would be the creation of a “diagnostic toolkit” consisting of a smartphone and a set of sensors and attachments that can be used to screen for a wide set of diseases in a community healthcare setting. However, there are a few concerns that still need to be overcome in low-cost diagnostics: lack of incentives to bring these devices to market, algorithmic bias, “black box” nature of the algorithms, and data storage/transfer concerns.

## Introduction

In medicine, physical measurements are crucial in assessing a patient’s health. The simplest measurements include weight, height, temperature, waist length, etc. These measurements, along with other vital signs and clinical observations, can provide valuable information to healthcare professionals as they diagnose and treat patients.

Some physical aspects of a disease are often difficult to discover without tools. A stethoscope is a primary tool that a doctor uses to screen for disease, and it is versatile in its ability to detect disease. The stethoscope relies on the amplification of internal sounds. For example, a clinician can use the stethoscope to identify valvular problems in the heart [1]. However, the stethoscope is limited in several ways: it cannot record audio over long periods of time, it cannot perform automatic analysis based on the audio (requires clinician expertise), and it only records audio data [2]. Healthcare workers would be empowered if they had a simple set of devices that can capture images, audio, texture/shape from touch, temperature, and electrochemical data from the skin surface with an accompanying system that can process that data. This set of tools would be ideal for screening of disease especially in under-resourced areas around the world where access to trained physicians, formal laboratory tests, and imaging are limited.

In this article, we describe what information can be gathered from simple physical measurements and recordings, outline some of the many of the diseases that could be diagnosed and monitored with these methods, and finally discuss how clinicians and engineers can effectively make these disease detection methods a reality.

## Low-cost and convenient methods

There are many ways to define “low-cost” for disease screening or diagnosis. In this work, we will use a definition that is not based on a set price. Instead, we are most interested in technologies that (1) can reduce the price of diagnosis in comparison to the current standard of care; or (2) can decrease long-term price of care through an early diagnosis method that is more accessible than the current gold standard. Some of the solutions presented in this article take advantage of smartphone technology since they satisfy both of the previous 2 conditions. Smartphones and sensor attachment for smartphones can be relatively cheap compared to other diagnostic tools (lab tests and imaging). They are also easy to transport and are becoming more available in lower resource environments [3,4]. In order to be considered “convenient,” the devices need to be portable and easy-to-use such that a minimally trained healthcare worker could collect information from a patient. In summary, the goal of this article is not to identify more accurate biological assays, higher sensitivity imaging techniques, or more convenient resource-intensive imaging techniques such X-ray or magnetic resonance imaging (MRI). Instead, we are trying to highlight ways in which simple sensors can be used to detect disease in a way that is more accessible.

## What physical data can be collected easily?

### Sound

Sound data is easy to collect and can be highly informative in diagnosing disease. Using a smartphone microphone or a separate small microphone, bodily sounds of the lung, heart, joints, and coughs can be used as a diagnostic tool for physicians [5–7]. An audio recording can be rapidly analyzed by software, and the results can then be electronically sent to a clinician without relying on an in-person visit. The effectiveness of collecting this data and analyzing it is dependent on whether the audio is of good enough quality to detect abnormalities. However, it is also possible for an application to clean the audio file to substantially reduce noise.

Stethoscopes and other analog medical diagnostic devices typically limit their frequency detection ranges from 20 Hz to 20 kHz, reflecting the detection range of human hearing. However, numerous medically relevant markers may fall beyond the limits of the human ear. Respiratory rates may fall to a fraction of a Hz [8], with amplitudes undetectable by human hearing [9]. Applications in seismocardiography have required frequency detection ranges from 1 to 30 Hz [10]. Digitized stethoscopes, accelerometers, and microelectromechanical systems have been designed to detect these signals, yielding novel medical diagnostic tools for identifying aberrant mechanical wave propagations across patient tissues [11–13]. Digitized techniques may also amplify sounds for improved audibility while filtering out obfuscating noises in clinical settings [14,15].

### Light

Like sound, light measurements (photography) could be useful in assessing a disease. Using built-in smartphone light production (flashlight) and analysis of incoming light (camera), useful information can be obtained. Other types of light measurements (e.g., ultraviolet and infrared) can be made through specific devices or by using attachments. These methods will be important in detecting fluorescence or pigmentation in certain areas of the body. Image-based diagnostics can be used to detect anemia, skin infections, skin cancer, vision loss, etc. [16–18].

Building on the foundational methods of light measurements, incorporating advanced imaging techniques such as hyperspectral imaging offers a more nuanced approach to disease screening. Hyperspectral imaging, which analyzes a wide spectrum of light, can significantly enhance the detection and analysis of various conditions by leveraging the unique spectral signatures of tissues [19]. Recent advances have made this methodology more accessible via a relatively low-cost smartphone attachment [20].

Additionally, the integration of sensor-based technologies such as pulse oximetry and optical blood pressure estimation further extends the scope of diagnostics using light. Pulse oximetry, for example, uses absorption principles to measure oxygen saturation in the blood, providing crucial information about respiratory and circulatory health [21]. Similarly, optical methods for estimating blood pressure offer a convenient alternative to traditional cuff-based measurements, potentially enabling continuous, real-time monitoring without direct physical contact [22]. These advancements underscore the potential of combining computerized analysis with light sensor data to augment traditional clinical assessments.

### Touch and kinematics

Numerous diseases present subtle but quantifiable changes in the motor function of patients. Videos of full-body motion, wearable sensors (placed on clothing and smartwatches), and drawing tasks are among some of the many approaches that have been proven to be useful in identifying disease [16,23–25]. Wearable sensors are particularly useful as they can provide longitudinal data from the patient and can be fitted with many different sensors including accelerometers, gyroscopes, thermometers, and pressure sensors. In addition, in the case of neurodegenerative diseases including Parkinson’s and Alzheimer’s disease, which can impact fine motor function and visuospatial processing, pen-tracking during drawing tasks have been used to identify disease [23,25].

Currently, gait analyses have been widely implemented as qualitative indicators of gross motor dysfunction. However, consistency in diagnosis and the ability to identify subtle abnormalities have limited the diagnostic capabilities of a clinician alone [26]. Recently, cheap sensors including accelerometers and inertial measurement units (IMUs), which measure 3D acceleration and rotational information, have been used for computerized gait analysis [27]. Sophisticated analysis of these data can make computerized gait analysis feasible and widely distributable to limited resourced settings.

### Electrical

Collecting electrical information is quite valuable as well. Headband electroencephalography (EEG) and electrocardiography (ECG) in wearable devices or in clothing can lead to important diagnoses [28,29]. It is well documented that neurological abnormalities may not be present all the time, especially during the limited time that a patient is in the clinic. Portable EEGs allow for longitudinal monitoring of gross brain activity. In addition, a portable ECG on a smartwatch may also be the only option to gain access to an ECG and can be used to quickly detect life-threatening arrhythmias and heart disease [30].

While smartwatch-based ECGs represent a significant advancement in making cardiac monitoring more accessible, it is important to recognize the inherent limitations of these devices due to their reliance on a single, distal lead. Such a configuration primarily allows for the detection of a subset of rhythm anomalies in addition to monitoring of heart rate [31,32]. Researchers continue to explore ways to enhance the diagnostic capabilities of wearable ECG technologies without compromising the devices’ portability and ease of use.

### Electrochemical

Electrical information from sweat can also be rich in information for diagnoses. Sensors can be placed on the skin to measure the electrical conductance, which correlates to the level of physiological activity (both physical and mental). Electrochemical data from sweat can provide useful information especially for the diagnosis of metabolic disorders [33]. Electrochemical data from interstitial fluids (ISF) can also provide biological information for detection of physical conditions. Previous studies that implemented wearable devices to collect electrochemical data from the ISF have successfully measured the concentration of transdermal alcohol and cortisol [34,35]. Other fluids such as urine and saliva have also provided useful electrochemical data that aided diagnosis of conditions. For example, lactic acid detection using salivary samples has been an important signal for hypoxia, whereas electrochemical information from urine was used to detect nitrite, which can hinder oxygen transports at high concentrations [36,37].

## How is the data used?

**Fig 1:**
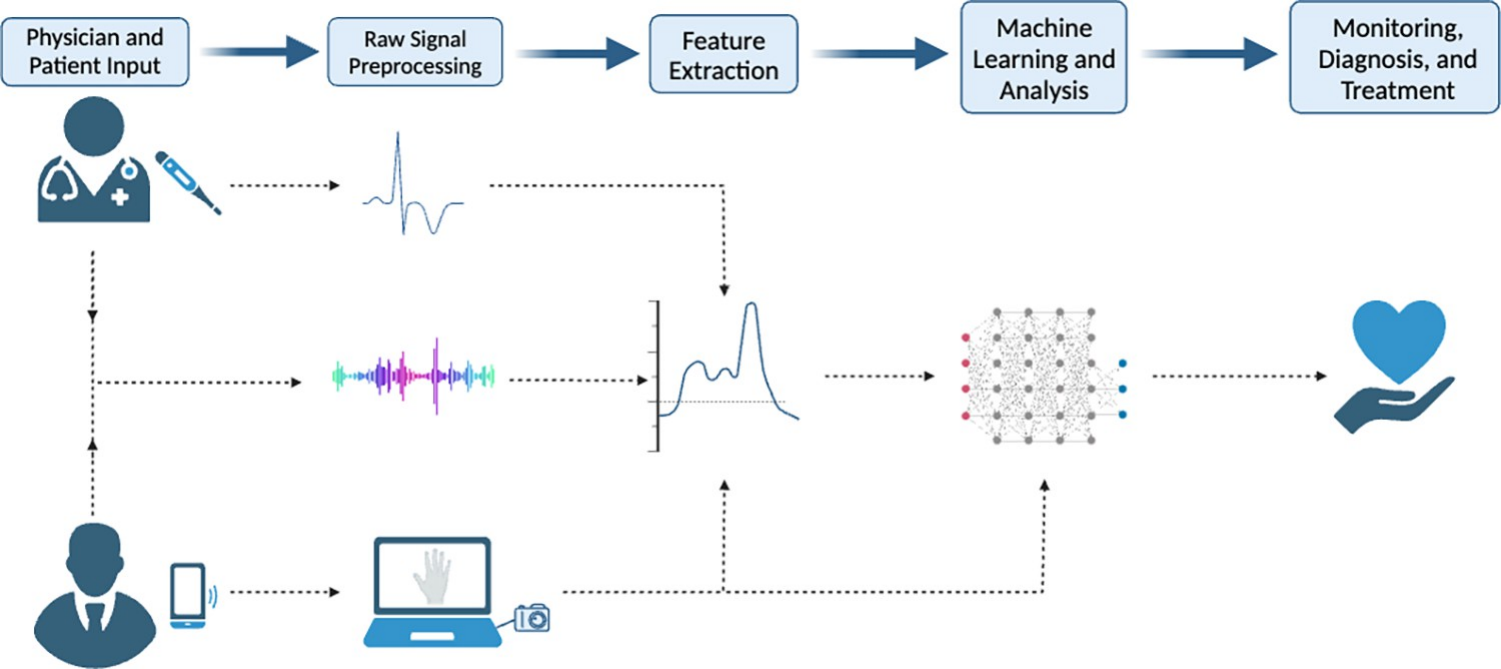
Data processing. This figure shows how the data is first collected either directly from the patient or from a healthcare worker. Features are extracted from the data to reduce the dimensionality for input into a machine learning model. The raw signal can also be directly inputted into a neural network for analysis. Lastly, the model provides a diagnosis, risk score, or disease severity that can be acted upon. Figure created with biorender.com.

Many of the approaches to detecting and monitoring diseases occur through similar pipelines (Fig 1). First, a raw signal is recorded by a camera, microphone, or other sensor. Then, important features are extracted from the raw signal. These features are fed into simple machine learning classifiers or regression models to predict a disease severity score or diagnosis. Another option is that the raw signal is fed into a neural network that will learn the important features directly.

## What conditions can be diagnosed and monitored using these techniques?

There are many implementations of low-cost medical technology that prove the potential of physical analysis of disease. Nearly every field of medicine can be improved by having a simple monitoring tool that can capture sound, light, electrical, touch, or kinematics data. S1 Table provides an extensive summary of many of the conditions, disorders, and diseases that can be diagnosed using low-cost methods that rely on simple measurements. We included neurological diseases, respiratory disorders, skin disorders, blood disorders, cardiac disorders, eye diseases, gastrointestinal disorders, geriatric conditions, musculoskeletal disorders, obstetrics disorders, and metabolic disorders.

Neurological diseases are particularly relevant since there are clear motor manifestations associated with these conditions. Alzheimer’s disease, Parkinson’s disease (and other movement disorders), Huntington’s disease, etc., all have motor manifestations that can be detected and quantified using drawing tests on low-cost tablets, eye tracking tests, and gait analysis [25,38,39]. In addition, recent algorithms have shown promise in detection of cardiac defects such as heart murmurs utilizing just a smartphone or digital stethoscope [40]. Smartphone microphones with accompanying algorithms have also been useful in detecting the presence of abnormal bowel sounds associated with irritable bowel syndrome with near perfect accuracy [7]. Additionally, a recent smartphone application was able to distinguish coughs from COVID-19 with coughs from other illnesses [41]. Furthermore, through simple pictures taken from a smartphone camera of the nail bed and blood smears, hemoglobin and sickle cell levels were able to be adequately detected respectively, assisting in the diagnosing of anemia and sickle cell disease [17,42]. Similarly, for eye disorders such as corneal disease, glaucoma, and diabetic retinopathy, a simple smartphone camera with portable attachments was able to identify the severity of these disorders [16,43,44].

## Longitudinal monitoring

Longitudinal physical measurements and recordings have become popular due to the wide use of smartwatches [45]. However, instead of just monitoring pulse, oxygen saturation, and temperature, a patient, for example, could have a small microphone or piezoelectric sensor placed on the abdomen or chest for suspected gastrointestinal or cardiac issues. Accelerometers or IMUs could be placed on limbs if there is suspicion of musculoskeletal or neurological problems. In addition, some signs of disease such as freezing of gait in patients with Parkinson’s disease are infrequent enough that they will not occur during an appointment [46]. However, recordings over a long period of time can pick up on this. Longitudinal monitoring does not just have to be passive. It can be active as well. Patients can be reminded to take pictures of their nail bed for anemia monitoring or they can do a quick drawing on their device to assess the effect of a medication to reduce tremors. In general, longitudinal monitoring can be done easily. The patient is given the device during the initial visit. The device collects data and analyzes it in real time. A health worker can monitor the results and then follow up with the patient at a later time. This framework has already been implemented for blood sugar monitoring.

## How to create low-cost diagnostic technologies

The background and recommendations provided here are a result of our work in developing low-cost medical technologies. With advances in low-cost sensors and smartphones, there is an opportunity to collect high-resolution information from patients that can then be automatically analyzed to provide patients with important diagnoses. These devices record simple data from the patient but use complex analysis methods in order to interpret the information. These devices should provide reasonably quick diagnoses and have the ability to operate with no dependence on the internet. This is so that they will be particularly useful in under-resourced settings where a disease screening tool could allow community health workers to provide care to patients. However, the tradeoffs related to the lack of internet in low-resource settings must be recognized. Studies have shown that areas without internet access have lower health outcomes than areas that do in part because of the inability to communicate urgent health findings to clinicians [47]. Additional resources must be put in place in efforts to make up for the lack of access to internet resources. Implementers of medical technologies in areas without the internet should prepare offline data recording methods or portable data storage devices with enough capacity to hold a large amount of medical data safely without access to cloud storage. In addition, depending on the type of model being used for data analysis and the quality of smartphone or portable computer being used, there may be a limitation in compute power. For example, running large neural networks on a mobile device may not be possible if the random access memory is not sufficient [48]. In addition, running large models on mobile devices will use significant battery power, which as an important consideration in low-resource settings [48].

There are a lot of opportunities to create diagnostic tools and treatments for limited resourced areas. We have observed 2 general types of models for doing the most important part of the creation of low-cost healthtech (need finding). (1) The inventors do extensive research into the health challenges in their region of interest. They read the literature and talk to experts, including the patients, in those regions. They then develop a technical solution to the problems. (2) Another option is to talk to clinicians and community health workers that have already identified a challenge and a potential solution [49]. The job of the inventor is to vet the feasibility of the solution, and then develop the technology. In this scheme, the clinicians or community health workers are equal partners with the inventors. Organizations like the Stanford Byers Center for Biodesign, Global Alliance for Medical Innovation at Harvard, and the Johns Hopkins Center for Bioengineering Innovation and Design use these general frameworks.

## Challenges and considerations in creating and deploying low-cost diagnostic technologies

We have reviewed many existing technologies that use smartphones or low-cost sensors to collect physical data and then process the simple data in a way that can aid in diagnosis of diseases. However, most of these methods have not been successfully deployed at scale. This may not be because of the shortcomings of the technology itself. There are numerous interrelated issues behind well-intentioned efforts to improve care delivery using technology in limited-resource settings that have been discussed previously [50]. One reason is that innovating in limited resourced areas is not as profitable [51]. Another explanation is that there is little incentive for scientists to deliver their promise of making healthcare more affordable. Their incentive is to publish. There needs to be a push by academic institutions to promote health equity as the goal of innovation instead of scientific recognition.

There are other real challenges in the deployment of these technologies. The medical community may not be ready for complex “black box” backend analysis [52]. Many of the innovations reviewed here employ complex feature extraction and deep learning to provide diagnosis or treatment recommendation. Importantly, the issues of data and algorithmic bias also need to be addressed to make sure that these technologies that leverage machine learning do not encode existing health disparities [53]. For example, a pulse oximeter has been shown to collect biased oxygen saturation readings in patients with darker skin, which leads to less oxygen administration for patients of color [54]. Even if the data collected is accurate, models can be biased if they aren’t trained on a representative population, which is a prevalent problem in current deep learning solutions for medicine [55]. In addition, many times when trying to increase the accessibility of a diagnostic technique, there will be a small decrease in accuracy of diagnosis due to the lower-resolution data collection method. Physicians may not find this decrease acceptable.

While devices may be relatively low-cost that does not necessarily mean that the method will cost less than having a community health care worker doing an in-person physical exam. There must be careful work done to ensure that these methods don’t increase patient costs when their goal is in fact to increase accessibility to healthcare. For example, it is possible that cloud storage of data can become expensive based on the storage service used especially as more data is collected. If one terabyte of storage is used to hold images after many months of collecting data, then depending on the service used, it could cost anywhere from $3–26/month depending on the type of data stored and the region the data is being stored in [56,57]. These costs will increase if the images need to be accessed frequently or if more data needs to be stored. While this is not very large cost, it must be factored in determining whether a new diagnostic test is cost effective. As mentioned in the previous section, we recommend running models on the device itself to ensure that additional costs are not incurred on top of data storage.

Furthermore, the regulatory landscape plays a crucial role in the deployment of these technologies. Regulatory agencies such as the US Food and Drug Administration (FDA) and the European Medicines Agency (EMA) have been starting to develop frameworks to evaluate the safety and effectiveness of machine learning-based diagnostic devices [58,59]. For example, the FDA has issued guidance on the clinical evaluation of software as a medical device, to ensure they meet rigorous safety and efficacy standards [60]. The evolving regulatory stance toward machine learning in diagnostics demands ongoing dialogue between innovators and regulators to balance innovation with patient safety and efficacy. The impact of these regulatory perspectives is profound, as they can either accelerate the deployment of effective, low-cost diagnostic technologies or hinder their progress [61].

Regardless, there has always been a gray area in the regulation of diagnostic medical technologies (especially those being tested) [62]. This means that there is a higher risk for leakage of health information collected through these new methods [63]. As a result, we recommend that inventors work extensively to ensure data storage/transfer security. Devices should not be deployed hastily due to the significant security considerations.

## The “Diagnostic Toolkit”

**Fig 2:**
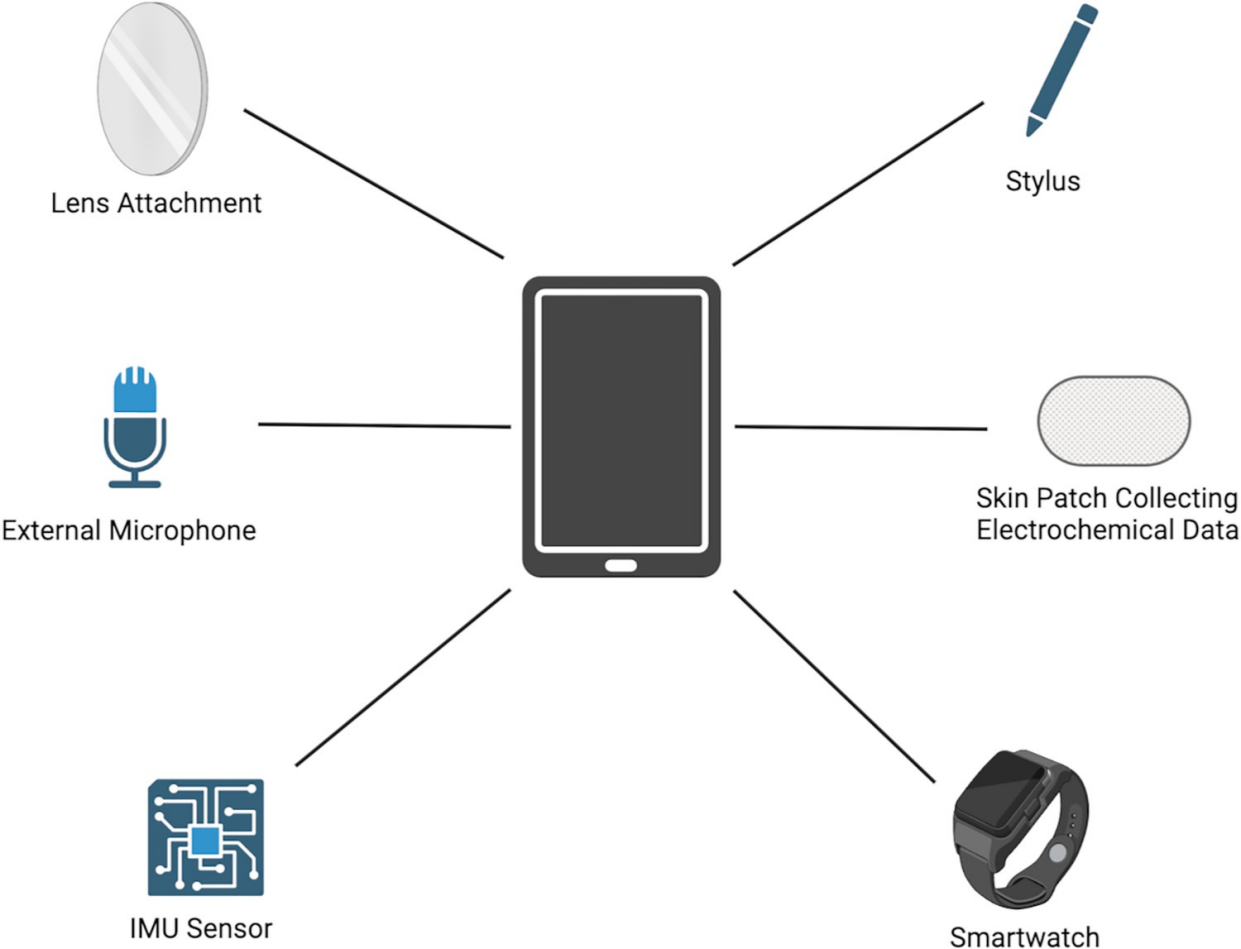
The Diagnostic Toolkit. This is an example of the possible components of a diagnostics toolkit. The centerpiece is a smartphone that can take pictures and receive data from the other sensors. Lens attachments can be used to visualize the eye and the skin better. A microphone can pick up breathing sounds and bowel sounds. IMUs or a smartwatch can monitor patient movements. A skin patch can communicate electrochemical information about sweat and hormone levels. Lastly, a stylus will be useful in collecting fine motor information based on patient drawings. Figure created with biorender.com.

Nevertheless, tools for diagnosis and monitoring that rely on simple physical recordings are promising. After a set of important and useful innovations have been identified, the ideal product would be one device or toolkit that combines many of the functionalities we have described (Fig 2). The toolkit may include IMUs and accelerometers that send data via Bluetooth to a smartphone. It may also include a piezoelectric sensor to amplify bowel sounds and another attachment that can amplify heart sounds. A set of lenses may also be in the kit to increase the magnification or alter the focal length of the camera on the smartphone. This will allow the camera to see details on the skin or the eye, for example. There may also be sensors to leave with the patients, so that their condition can be monitored over time. For example, a skin patch that monitors cortisol or alcohol levels can be left for the patient to wear throughout the day. The goal is to have an easy-to-use method to diagnose and monitor disease when expensive diagnostic equipment is not available. But this goal can only be realized in an innovation ecosystem that promotes collaboration, capacity-building, and co-creation that includes those who are disproportionately burdened by disease.

## Conclusions

Given the increased availability of cheap sensors and smartphones globally in recent years, doing relatively low-cost screening or diagnosis of disease has become feasible. Research into these techniques has yielded many promising diagnostic tools spanning most fields of medicine from neurological diseases to musculoskeletal diseases to cardiac disorders. Only a few of these methods have actually impacted clinical care in part due to the lack of financial incentive to innovate in the low-cost medical devices. We also recognize that there are concerns with these technologies including the “black box” nature of some of the algorithms used, the security concerns for data transfer and storage, and the possibly lower accuracy compared to the gold standard. However, these limitations can be partially overcome with careful model creation and security measures. The ultimate goal is to have a “diagnostic toolkit” that combines many of the most important tools. This can then be deployed in a hospital setting or more importantly in a resource-limited community healthcare setting.

## Supporting information

S1 Table Screening of Medical Disorders using Low-Cost Methods. This table provides the target condition of an automated or digitized diagnostic tool which has been developed in previous studies. In many cases, the diagnosis of these conditions early in disease progression benefits patients significantly through timely interventions and targeted monitoring. However, for these conditions, many of which are common or growing increasingly so, the costs of screening large populations may overburden limited physician resources. The table aims to present proof of concepts for low-cost diagnosis and monitoring of disease through the collection of physical data from various sensors. Furthermore, the table provides the accuracies achieved in previous studies using these techniques and offers context in the form of minimum equipment lists required to implement these methods. Lastly, the table presents comparisons to the generally accepted diagnostic technique. The disorders covered here are classified into respiratory, blood, cardiovascular, eye, gastrointestinal, neurological, geriatrics, musculoskeletal, skin, mitochondrial, and obstetrics.
(DOCX)

## References

[1] Padilla-Ortiz AL, Ibarra D. Lung and Heart Sounds Analysis: State-of-the-Art and Future Trends. Crit Rev Biomed Eng. 2018;46(1):33–52. doi: 10.1615/CritRevBiomedEng.2018025112 29717676

[2] Seah JJ, Zhao J, Wang DY, Lee HP. Review on the Advancements of Stethoscope Types in Chest Auscultation. Diagnostics (Basel). 2023 Apr 25;13(9):1545. doi: 10.3390/diagnostics13091545 37174938 PMC10177339

[3] Poushter J, Caldwell B, Chwe Hanyu. 2. Smartphone ownership on the rise in emerging economies [Internet]. Pew Research Center’s Global Attitudes Project. 2018 [cited 2024 Apr 4]. Available from: https://www.pewresearch.org/global/2018/06/19/2-smartphone-ownership-on-the-rise-in-emerging-economies/.

[4] McCool J, Dobson R, Whittaker R, Paton C. Mobile Health (mHealth) in Low- and Middle-Income Countries. Annu Rev Public Health. 2022 Apr 5;43(Volume 43, 2022):525–39. doi: 10.1146/annurev-publhealth-052620-093850 34648368

[5] Yeginer M, Ciftci K, Cini U, Sen I, Kilinc G, Kahya YP. Using lung sounds in classification of pulmonary diseases according to respiratory subphases. In: The 26th Annual International Conference of the IEEE Engineering in Medicine and Biology Society. 2004. p. 482–5. doi: 10.1109/IEMBS.2004.1403199 17271718

[6] Cao M, Gardner RS, Hariharan R, Nair DG, Schulze C, An Q, et al. Ambulatory Monitoring of Heart Sounds via an Implanted Device Is Superior to Auscultation for Prediction of Heart Failure Events. J Card Fail. 2020 Feb 1;26(2):151–9. doi: 10.1016/j.cardfail.2019.10.006 31634574

[7] Kutsumi Y, Kanegawa N, Zeida M, Matsubara H, Murayama N. Automated Bowel Sound and Motility Analysis with CNN Using a Smartphone. Sensors (Basel). 2022 Dec 30;23(1):407. doi: 10.3390/s23010407 36617005 PMC9824196

[8] Pasterkamp H, Kraman SS, Wodicka GR. Respiratory Sounds. Am J Respir Crit Care Med. 1997 Sep;156(3):974–87. 9310022 10.1164/ajrccm.156.3.9701115

[9] Rossing T. Springer Handbook of Acoustics. Springer Science & Business Media; 2007. p. 1179.

[10] Liu Y, Norton JJS, Qazi R, Zou Z, Ammann KR, Liu H, et al. Epidermal mechano-acoustic sensing electronics for cardiovascular diagnostics and human-machine interfaces. Sci Adv. 2016 Nov 16;2(11):e1601185. doi: 10.1126/sciadv.1601185 28138529 PMC5262452

[11] Okamoto Y, Nguyen TV, Takahashi H, Takei Y, Okada H, Ichiki M. Highly sensitive low-frequency-detectable acoustic sensor using a piezoresistive cantilever for health monitoring applications. Sci Rep. 2023 Apr 20;13(1):6503. doi: 10.1038/s41598-023-33568-3 37081122 PMC10119305

[12] Ha T, Tran J, Liu S, Jang H, Jeong H, Mitbander R, et al. A Chest-Laminated Ultrathin and Stretchable E-Tattoo for the Measurement of Electrocardiogram, Seismocardiogram, and Cardiac Time Intervals. Adv Sci (Weinh). 2019 Jul 17;6(14):1900290. doi: 10.1002/advs.201900290 31380208 PMC6662084

[13] Zhang S, Zhang R, Chang S, Liu C, Sha X. A Low-Noise-Level Heart Sound System Based on Novel Thorax-Integration Head Design and Wavelet Denoising Algorithm. Micromachines. 2019 Dec;10(12):885. doi: 10.3390/mi10120885 31861068 PMC6953004

[14] Aumann HM, Emanetoglu NW. Stethoscope with digital frequency translation for improved audibility. Healthc Technol Lett. 2019 Jul 31;6(5):143–6. doi: 10.1049/htl.2019.0011 31839970 PMC6863143

[15] McLane I, Emmanouilidou D, West JE, Elhilali M. Design and Comparative Performance of a Robust Lung Auscultation System for Noisy Clinical Settings. IEEE J Biomed Health Inform. 2021 Jul;25(7):2583–94. doi: 10.1109/JBHI.2021.3056916 33534721 PMC8374873

[16] Mantena S, Chandra J, Pecyna E, Zhang A, Garrity D, Ong Tone S, et al. Low-Cost, Smartphone-Based Specular Imaging and Automated Analysis of the Corneal Endothelium. Transl Vis Sci Technol. 2021 Apr 2;10(4):4. doi: 10.1167/tvst.10.4.4 34003981 PMC8024782

[17] Mannino RG, Myers DR, Tyburski EA, Caruso C, Boudreaux J, Leong T, et al. Smartphone app for non-invasive detection of anemia using only patient-sourced photos. Nat Commun. 2018 Dec 4;9(1):4924. doi: 10.1038/s41467-018-07262-2 30514831 PMC6279826

[18] Stenum J, Rossi C, Roemmich RT. Two-dimensional video-based analysis of human gait using pose estimation. PLoS Comput Biol. 2021 Apr 23;17(4):e1008935. doi: 10.1371/journal.pcbi.1008935 33891585 PMC8099131

[19] Yoon J. Hyperspectral Imaging for Clinical Applications. Biochip J. 2022 Mar 1;16(1):1–12.

[20] Stuart MB, McGonigle AJS, Davies M, Hobbs MJ, Boone NA, Stanger LR, et al. Low-Cost Hyperspectral Imaging with A Smartphone. J Imaging. 2021 Aug 5;7(8):136. doi: 10.3390/jimaging7080136 34460772 PMC8404918

[21] Wick KD, Matthay MA, Ware LB. Pulse oximetry for the diagnosis and management of acute respiratory distress syndrome. Lancet Respir Med. 2022 Nov;10(11):1086–98. doi: 10.1016/S2213-2600(22)00058-3 36049490 PMC9423770

[22] Luo H, Yang D, Barszczyk A, Vempala N, Wei J, Wu SJ, et al. Smartphone-Based Blood Pressure Measurement Using Transdermal Optical Imaging Technology. Circ Cardiovasc Imaging. 2019;12(8):e008857. 31382766 10.1161/CIRCIMAGING.119.008857

[23] Chandra J, Muthupalaniappan S, Shang Z, Deng R, Lin R, Tolkova I, et al. Screening of Parkinson’s Disease Using Geometric Features Extracted from Spiral Drawings. Brain Sci. 2021 Oct;11(10):1297. doi: 10.3390/brainsci11101297 34679363 PMC8533717

[24] De Vos M, Prince J, Buchanan T, FitzGerald JJ, Antoniades CA. Discriminating progressive supranuclear palsy from Parkinson’s disease using wearable technology and machine learning. Gait Posture. 2020 Mar;77:257–63. doi: 10.1016/j.gaitpost.2020.02.007 32078894

[25] Luciano MS, Wang C, Ortega RA, Yu Q, Boschung S, Soto-Valencia J, et al. Digitized Spiral Drawing: A Possible Biomarker for Early Parkinson’s Disease. PLoS ONE. 2016 Oct 12;11(10):e0162799. doi: 10.1371/journal.pone.0162799 27732597 PMC5061372

[26] Buckley C, Alcock L, McArdle R, Rehman RZU, Del Din S, Mazzà C, et al. The Role of Movement Analysis in Diagnosing and Monitoring Neurodegenerative Conditions: Insights from Gait and Postural Control. Brain Sci. 2019 Feb 6;9(2):34. doi: 10.3390/brainsci9020034 30736374 PMC6406749

[27] Mobbs RJ, Perring J, Raj SM, Maharaj M, Yoong NKM, Sy LW, et al. Gait metrics analysis utilizing single-point inertial measurement units: a systematic review. Mhealth. 2022 Jan 20;8:9. doi: 10.21037/mhealth-21-17 35178440 PMC8800203

[28] Arnal PJ, Thorey V, Debellemaniere E, Ballard ME, Bou Hernandez A, Guillot A, et al. The Dreem Headband compared to polysomnography for electroencephalographic signal acquisition and sleep staging. Sleep. 2020 May 20;43(11):zsaa097. doi: 10.1093/sleep/zsaa097 32433768 PMC7751170

[29] Li M, Xiong W, Li Y. Wearable Measurement of ECG Signals Based on Smart Clothing. Int J Telemed Appl. 2020 Jan 18;2020:6329360. doi: 10.1155/2020/6329360 32395127 PMC7201832

[30] Caillol T, Strik M, Ramirez FD, Abu-Alrub S, Marchand H, Buliard S, et al. Accuracy of a Smartwatch-Derived ECG for Diagnosing Bradyarrhythmias, Tachyarrhythmias, and Cardiac Ischemia. Circ Arrhythm Electrophysiol. 2021 Jan;14(1):e009260. doi: 10.1161/CIRCEP.120.009260 33441002

[31] Mitchell H, Rosario N, Hernandez C, Lipsitz SR, Levine DM. Single-lead arrhythmia detection through machine learning: cross-sectional evaluation of a novel algorithm using real-world data. Open Heart. 2023 Sep 20;10(2):e002228. doi: 10.1136/openhrt-2022-002228 37734747 PMC10514635

[32] Inui T, Kohno H, Kawasaki Y, Matsuura K, Ueda H, Tamura Y, et al. Use of a Smart Watch for Early Detection of Paroxysmal Atrial Fibrillation: Validation Study. JMIR Cardio. 2020 Jan 22;4(1):e14857. doi: 10.2196/14857 32012044 PMC7003123

[33] Gao F, Liu C, Zhang L, Liu T, Wang Z, Song Z, et al. Wearable and flexible electrochemical sensors for sweat analysis: a review. Microsyst Nanoeng. 2023 Jan 1;9(1):1–21. doi: 10.1038/s41378-022-00443-6 36597511 PMC9805458

[34] Venugopal M, Feuvrel KE, Mongin D, Bambot S, Faupel M, Panangadan A, et al. Clinical Evaluation of a Novel Interstitial Fluid Sensor System for Remote Continuous Alcohol Monitoring. IEEE Sens J. 2008 Jan;8(1):71–80.

[35] Venugopal M, Arya SK, Chornokur G, Bhansali S. A realtime and continuous assessment of cortisol in ISF using electrochemical impedance spectroscopy. Sens Actuators A Phys. 2011 Dec 1;172(1):154–60. doi: 10.1016/j.sna.2011.04.028 22163154 PMC3234992

[36] Malon RSP, Sadir S, Balakrishnan M, Córcoles EP. Saliva-Based Biosensors: Noninvasive Monitoring Tool for Clinical Diagnostics. Biomed Res Int. 2014;2014:962903. doi: 10.1155/2014/962903 25276835 PMC4172994

[37] Zhou Y, Ma M, He H, Cai Z, Gao N, He C, et al. Highly sensitive nitrite sensor based on AuNPs/RGO nanocomposites modified graphene electrochemical transistors. Biosens Bioelectron. 2019 Dec 15;146:111751. doi: 10.1016/j.bios.2019.111751 31605988

[38] Hicks SL, Robert MPA, Golding CVP, Tabrizi SJ, Kennard C. Chapter 6.9—Oculomotor deficits indicate the progression of Huntington’s Disease. In: Kennard C, Leigh RJ, editors. Progress in Brain Research [Internet]. Elsevier; 2008 [cited 2023 May 27]. p. 555–8. (Using Eye Movements as an Experimental Probe of Brain Function; vol. 171). Available from: https://www.sciencedirect.com/science/article/pii/S007961230800678X. 10.1016/S0079-6123(08)00678-X 18718352

[39] Nonnekes J, Růžička E, Serranová T, Reich SG, Bloem BR, Hallett M. Functional gait disorders: A sign-based approach. Neurology. 2020 Jun 16;94(24):1093–9. doi: 10.1212/WNL.0000000000009649 32482839 PMC7455329

[40] Chorba JS, Shapiro AM, Le L, Maidens J, Prince J, Pham S, et al. Deep Learning Algorithm for Automated Cardiac Murmur Detection via a Digital Stethoscope Platform. J Am Heart Assoc. 2021 May 4;10(9):e019905. doi: 10.1161/JAHA.120.019905 33899504 PMC8200722

[41] Imran A, Posokhova I, Qureshi HN, Masood U, Riaz MS, Ali K, et al. AI4COVID-19: AI enabled preliminary diagnosis for COVID-19 from cough samples via an app. Inform Med Unlocked. 2020;20:100378. doi: 10.1016/j.imu.2020.100378 32839734 PMC7318970

[42] Ilyas S, Sher M, Du E, Asghar W. Smartphone-based Sickle Cell Disease Detection and Monitoring for Point-of-Care Settings. Biosens Bioelectron. 2020 Oct 1;165:112417. doi: 10.1016/j.bios.2020.112417 32729535 PMC7484220

[43] Effective Low-Cost Ophthalmological Screening With a Novel iPhone Fundus Camera at Community Centers [Internet]. Cureus. [cited 2023 May 13]. Available from: https://www.cureus.com/articles/106024-effective-low-cost-ophthalmological-screening-with-a-novel-iphone-fundus-camera-at-community-centers. 10.7759/cureus.28121 PMC9389029 35990564

[44] Stratton S, Luna J, Roh S, Shaikh N, Alwreikat A, Jiang Y, et al. Smartphone-based Fundus Photography for Remote Glaucoma Assessment in a Low-Resource Setting. Invest Ophthalmol Vis Sci. 2021 Jun 21;62(8):1616.

[45] Reeder B, David A. Health at hand: A systematic review of smart watch uses for health and wellness. J Biomed Inform. 2016 Oct 1;63:269–76. doi: 10.1016/j.jbi.2016.09.001 27612974

[46] Mancini M, Smulders K, Cohen RG, Horak FB, Giladi N, Nutt JG. The Clinical Significance Of Freezing While Turning in Parkinson’s Disease. Neuroscience. 2017 Feb 20;343:222–8. doi: 10.1016/j.neuroscience.2016.11.045 27956066 PMC5289743

[47] Yu J, Meng S. Impacts of the Internet on Health Inequality and Healthcare Access: A Cross-Country Study. Front Public Health. 2022 Jun 9;10:935608. doi: 10.3389/fpubh.2022.935608 35757602 PMC9218541

[48] Benedetto JI, Sanabria P, Neyem A, Navon J, Poellabauer C, Xia B Ning Deep Neural Networks on Mobile Healthcare Applications: Practical Recommendations. Proceedings. 2018;2(19):550.

[49] Process [Internet]. Stanford Byers Center for Biodesign. [cited 2024 Apr 6]. Available from: https://biodesign.stanford.edu/about-us/process.html.

[50] DePasse J, Celi LA. Collaboration, capacity building and co-creation as a new mantra in global health. International J Qual Health Care. 2016 Sep 1;28(4):536–7. doi: 10.1093/intqhc/mzt077 24225268

[51] Diaconu K, Chen YF, Manaseki-Holland S, Cummins C, Lilford R. Medical device procurement in low- and middle-income settings: protocol for a systematic review. Syst Rev. 2014 Oct 21;3(1):118. doi: 10.1186/2046-4053-3-118 25336161 PMC4211929

[52] Wilkinson J, Arnold KF, Murray EJ, van Smeden M, Carr K, Sippy R, et al. Time to reality check the promises of machine learning-powered precision medicine. Lancet Digit Health. 2020 Dec;2(12):e677–80. doi: 10.1016/S2589-7500(20)30200-4 33328030 PMC9060421

[53] Celi LA, Cellini J, Charpignon ML, Dee EC, Dernoncourt F, Eber R, et al. Sources of bias in artificial intelligence that perpetuate healthcare disparities—A global review. PLoS Digit Health. 2022 Mar 31;1(3):e0000022. doi: 10.1371/journal.pdig.0000022 36812532 PMC9931338

[54] Gottlieb ER, Ziegler J, Morley K, Rush B, Celi LA. Assessment of Racial and Ethnic Differences in Oxygen Supplementation Among Patients in the Intensive Care Unit. JAMA Intern Med. 2022 Aug 1;182(8):849–58. doi: 10.1001/jamainternmed.2022.2587 35816344 PMC9274443

[55] Kaushal A, Altman R, Langlotz C. Geographic Distribution of US Cohorts Used to Train Deep Learning Algorithms. JAMA. 2020 Sep 22;324(12):1212–3. doi: 10.1001/jama.2020.12067 32960230 PMC7509620

[56] Pricing | Cloud Healthcare API [Internet]. Google Cloud. [cited 2024 Apr 6]. Available from: https://cloud.google.com/healthcare-api/pricing.

[57] Medical Imaging in the Cloud–AWS HealthImaging Pricing–AWS [Internet]. Amazon Web Services, Inc. [cited 2024 Apr 6]. Available from: https://aws.amazon.com/healthimaging/pricing/.

[58] Benjamens S, Dhunnoo P, Meskó B. The state of artificial intelligence-based FDA-approved medical devices and algorithms: an online database. NPJ Digit Med. 2020 Sep 11;3:118. doi: 10.1038/s41746-020-00324-0 32984550 PMC7486909

[59] Hines PA, Guy RH, Humphreys AJ, Papaluca-Amati M. The European Medicines Agency’s goals for regulatory science to 2025. Nat Rev Drug Discov. 2019 Jun;18(6):403–4. doi: 10.1038/d41573-019-00071-2 31160761

[60] Health C for D and R. Artificial Intelligence and Machine Learning in Software as a Medical Device. FDA [Internet]. 2024 Mar 15 [cited 2024 Apr 4]. Available from: https://www.fda.gov/medical-devices/software-medical-device-samd/artificial-intelligence-and-machine-learning-software-medical-device.

[61] Institute of Medicine (US) Committee on the Public Health Effectiveness of the FDA 510(k) Clearance Process, Wizemann T. Impact of the Regulatory Framework on Medical Device Development and Innovation. In: Public Health Effectiveness of the FDA 510(k) Clearance Process: Balancing Patient Safety and Innovation: Workshop Report [Internet]. National Academies Press (US); 2010 [cited 2024 Apr 6]. Available from: https://www.ncbi.nlm.nih.gov/books/NBK209794/. 24983045

[62] Carter A, Liddle J, Hall W, Chenery H. Mobile Phones in Research and Treatment: Ethical Guidelines and Future Directions. JMIR Mhealth Uhealth. 2015 Oct 16;3(4):e4538. doi: 10.2196/mhealth.4538 26474545 PMC4704925

[63] Larson RS. A Path to Better-Quality mHealth Apps. JMIR Mhealth Uhealth. 2018 Jul 30;6(7):e10414. doi: 10.2196/10414 30061091 PMC6090170

