# Supplementary material for: Low-cost and convenient screening of disease using analysis of physical measurements and recordings
Source: PLOS Digit Health. 2024 Sep 19;3(9):e0000574. doi: 10.1371/journal.pdig.0000574 (PMC11412657; doi:10.1371/journal.pdig.0000574)
Supplement: S1 Table — This table provides the target condition of an automated or digitized diagnostic tool which has been developed in previous studies. In many cases, the diagnosis of these conditions early in disease progression benefits patients significantly through timely interventions and targeted monitoring. However, for these conditions, many of which are common or growing increasingly so, the costs of screening large populations may overburden limited physician resources. The table aims to present proof of concepts for low-cost diagnosis and monitoring of disease through the collection of physical data from various sensors. Furthermore, the table provides the accuracies achieved in previous studies using these techniques and offers context in the form of minimum equipment lists required to implement these methods. Lastly, the table presents comparisons to the generally accepted diagnostic technique. The disorders covered here are classified into respiratory, blood, cardiovascular, eye, gastrointestinal, neurological, geriatrics, musculoskeletal, skin, mitochondrial, and obstetrics. (DOCX) [file pdig.0000574.s001.docx]

**S1 Table.** **Screening of Medical Disorders using Low-Cost Methods**. This table provides the target condition of an automated or digitized diagnostic tool which has been developed in previous studies. In many cases, the diagnosis of these conditions early in disease progression benefits patients significantly through timely interventions and targeted monitoring. However, for these conditions, many of which are common or growing increasingly so, the costs of screening large populations may overburden limited physician resources. The table aims to present proof of concepts for low-cost diagnosis and monitoring of disease through the collection of physical data from various sensors. Furthermore, the table provides the accuracies achieved in previous studies using these techniques and offers context in the form of minimum equipment lists required to implement these methods. Lastly, the table presents comparisons to the generally accepted diagnostic technique. The disorders covered here are classified into respiratory, blood, cardiovascular, eye, gastrointestinal, neurological, geriatrics, musculoskeletal, skin, mitochondrial, and obstetrics.

| **Target Condition** | **Detection Method** | **Analysis Methods** | **Accuracy of the Method** | **Minimum Equipment Required** | **Gold Standard and its accuracy** | **Supporting Papers** |
| --- | --- | --- | --- | --- | --- | --- |
| **Respiratory Disorders** | | | | | | |
| Chronic obstructive pulmonary disease (COPD): an umbrella term for several illnesses that result in the blockage of airways and the onset of respiratory issues. COPD encompasses conditions like chronic bronchitis and emphysema. | To detect COPD, the vibrations of the chest wall can be recorded using a small and sensitive sensor. Condenser microphones would make an ideal sensor as they provide both of these attributes, but other types of microphones can still be used. There are other components that can be added to noise and filter out other sounds (from the heart). | The raw signals from the microphone can be used in many ways. One way is to calculate various features relating to the distribution of the sound signal. Another is to calculate features related to the frequency spectrum of the sounds. Lastly, one could pre-process the sound signal and feed it into a neural network for direct classification. | This method has shown varying accuracy but using neural networks, this method has a 72% accuracy | Microphone | Spirometry (measurement of inhalation and exhalation volume) is the gold standard method of diagnosis and its accuracy is around 90%. | Yeginer et al 2005[^1^](https://www.zotero.org/google-docs/?7xPJqN), Reichert et al 2008[^2^](https://www.zotero.org/google-docs/?rSGryq) |
| Asthma: a condition that results in the narrowing and swelling of airways, which may cause mucus production that impedes breathing, producing a whistling noise known as wheezing and prompting shortness of breath. | An electrochemical sensor detects nitrite content in exhaled breath condensate. Nitrite content in breath has been linked to asthma and other respiratory conditions. | The measured nitrite content is directly used in detecting asthma and potentially the severity of asthma. | The sensor has a detection limit of 830nM and has a sensitivity of 0.1 μA μM^-1^ cm^-2^ | Small graphene sensor and breath condenser | Spirometry (measurement of inhalation and exhalation volume) is the gold standard. Its sensitivity is much lower than for COPD. Exhaled nitric oxide tests are also used. | Gholizadeh et al 2017, Saglani et al 2019[^4^](https://www.zotero.org/google-docs/?S2RRRI) |
| COVID-19: a respiratory illness that is caused by SARS-CoV-2. | A group of researchers at the University of Cambridge developed an application that can effectively diagnose COVID-19 using a recording of the patient’s coughs. Patients were asked to “cough three times”, to “breathe deeply through their mouth three to five times”, and to “read a short sentence appearing on the screen three times”. | The auditory features of the recording, such as duration, onset, and tempo were extracted and the researchers utilized these features to distinguish COVID-19 patients from healthy control patients. | The researchers showed AUC values of up to 0.82 in these distinguishing tasks between controls and patients. It was able to detect coughs 96% of the time. Of the coughs detected, it was able to distinguish between COVID-19 or another illness 89% of the time. | Smartphone Microphone | The current method to diagnose COVID-19 is through PCR testing, which replicates viral particles and identifies them based on their size and interactions. PCR can have approximately a clinical 95% specificity and 80% sensitivity. | Imran et al 2020[^5^](https://www.zotero.org/google-docs/?m8ZGRq), Brown et al 2021[^6^](https://www.zotero.org/google-docs/?TB0PZr) |
| **Blood Disorders** | | | | | | |
| Anemia: a condition that occurs when one’s blood doesn’t have the necessary number of healthy red blood cells. The lack of red blood cells leads to reduced oxygen flow in the body. | A smart device is used to take a picture of a patient’s finger bed. It then focuses on the fingernails of the patient. Using flash from the image as a baseline for color correction, it is able to detect abnormalities in the finger bed and predict hemoglobin levels from such abnormalities. | Fingernail, skin color, and image metadata were extracted from each picture of the patient’s finger bed. A linear regression model was created between these variables and hemoglobin values to yield a predictive model of hemoglobin from pictures of the finger bed. | Hemoglobin level was detected within ±2.4 g dL^-1^ accuracy, and a sensitivity for anemia detection of 97%. | Smart device with camera | The standard for detecting anemia is to order a Complete Blood Count test (or CBC). This is extremely accurate and measures the proportion of red blood cells in blood and hemoglobin in blood. | Mannino et al 2018[^7^](https://www.zotero.org/google-docs/?LTBU62) |
| Sickle cell disease: a group of disorders which causes red blood cells to distort into a sickle-like shape. This genetic condition leads to early red blood cell death, which ultimately leads to anemia. | To detect sickle cell, a smart device is able to take a picture of a blood smear. The researchers attached a simple lens to the smartphone camera, developed a piece of hardware to hold the microfluidic chip, and placed a source of light under the slide. | A classical image processing algorithm is used to segment the image into individual red blood cells and then count the non-circular cells. | Results for detecting sickle cell were fairly accurate, with detection not deviating any more than 7% from the true value of sickling. | Smart device with camera, 3D printed accessory | The standard for diagnosing sickle cell is a blood test and microscope screening for defective hemoglobin. | Ilyas et al 2020[^8^](https://www.zotero.org/google-docs/?Z6NYih) |
| **Cardiovascular Disorders** | | | | | | |
| Heart Murmur: sounds caused by abnormal turbulent sounds of blood flowing through the heart. This is usually due to diseased or degrading heart valves. | Researchers drew upon more than thirty-four hours of heart sound audio recordings from digital stethoscopes. | These researchers created a convolutional neural network (CNN) to identify clinically significant heart murmurs and valve dysfunction from the audio data. The CNN outputs whether there is a heart murmur or not. | Algorithm performance for detecting murmurs has sensitivity and specificity of 76.3% and 91.4%, respectively. Omitting murmurs with grade 1 intensity, the sensitivity increased to 90.0%. | Microphone (this study used a digital stethoscope) | Heart murmurs are traditionally identified by health care professionals with stethoscopes and through echocardiograms. | Chorba et al 2021[^9^](https://www.zotero.org/google-docs/?Sgkya9) |
| Heart Failure: a well-diagnosed and complicated syndrome characterized with a third heart sound (S3) that occurs during the early diastolic phase of passive blood transportation into the ventricles. | Health care professionals were able to use an implanted microphone to evaluate their predictive capabilities for HF. Once implanted, it would be able to detect the third heart sound (S3), the early diastolic phase of passive blood movement into ventricles as a predictor for HF. | A set of filters were applied to raw heart sound waveforms collected from the implanted device. A proprietary algorithm was then used to identify the S3 heart sound. | The prognostic power of the device was better than normal auscultations both for follow-up visits (Hazard Ratio = 5.7) and over the whole study (Hazard Ratio = 2.9). | Skin-deep implanted microphone | Currently, there is no standard way to diagnose heart failure. Doctors typically look at a patient's medical history, symptoms and perform a physical examination including cardiac auscultation | Cao et al 2019[^10^](https://www.zotero.org/google-docs/?aJ6wdl) |
| Heart Failure:  (see above) | Using just a microphone, doctors were able to analyze heart sounds. The microphone could be placed at either the aortic area, the pulmonic area, the tricuspid area, and the mitral area. | Based on a number of different time and frequency-based features, an algorithm then analyzes if the patient is predisposed to heart failure. | Using those features, they were able to analyze compensated and decompensated phases with an accuracy of 93.2% | Smartphone microphone | (See above) | Gjoreski et al 2020[^11^](https://www.zotero.org/google-docs/?pQbTvf)  Kang et al 2018[^12^](https://www.zotero.org/google-docs/?F0yOZd) |
| **Eye Disorders** | | | | | | |
| Diabetic Retinopathy: an impairment of the retina that leads to losses of vision. Diabetic retinopathy is a complication of diabetes that results from the damage of the blood vessels of the retina caused by the high blood sugar levels | A smartphone camera assisted technology is being developed for imaging the eye to diagnose retinopathy. It relies on a variety of attachments and lenses to get an image with usable quality. | DeepDR is a deep-learning system created to evaluate retinal image quality, retinal lesions, and DR grades in order to detect Diabetic Retinopathy. DR severity was graded into five levels (non-DR, mild NPDR, moderate NPDR, severe NPDR, or PDR, respectively), according to the International Clinical Diabetic Retinopathy Disease Severity Scale. | The average AUC is 0.96 for DR grading | Smartphone, lenses, and DeepDR | A clinician grades fundus images. DR grades (non-DR, mild NPDR, moderate NPDR, severe NPDR, or PDR) are used. | Dai et al 2021[^13^](https://www.zotero.org/google-docs/?9PISVs), Tan et al 2019[^14^](https://www.zotero.org/google-docs/?J9Aptg) |
| Glaucoma: a group of eye conditions that causes blindness due to the damage of the optic nerve secondary to high intraocular pressure. | A smartphone camera attached to the PanOptic iExaminer was utilized to take a photograph of the eye at low resource settings. | The cup-to-disc ratio (CDR) from the stereoscopic images were calculated manually. The diagnosis of glaucoma using the CDR ratio, conducted in a non-clinical setting was compared to the diagnosis of glaucoma by clinicians using the image taken. | The cup-to-disc ratio (CDR) judged from the stereoscopic images showed moderate reliability and good agreement with the CDR reported by the treating ophthalmologists (ICC 0.59; r = 0.792, p < 0.001). | A smartphone camera attached to a PanOptic iExaminer | The cup-to-disc ratio (CDR) is one method although intraocular pressure and visual field tests are also done. | Stratton et al 2021[^15^](https://www.zotero.org/google-docs/?7T6fAu), Idriss et al 2021[^16^](https://www.zotero.org/google-docs/?Es1zZJ) |
| Corneal endotheliopathy or corneal endothelial dysfunction: a reduction of the integrity at the level of the corneal endothelium. Primary corneal endotheliopathy can be caused by conditions such as Fuchs endothelial corneal dystrophy (FECD) and secondary endotheliopathy can be caused due to intraocular surgery, such as cataract surgery. | A slit lamp microscope that is attachable to the smartphone camera can be used to monitor the corneal epithelium. The smartphone takes the image and the slit lamp is used to provide extra magnification and focus. | Classical image processing techniques were used to segment the cells and then calculate relevant metrics of corneal endothelial health: endothelial cell density (ECD), percentage of hexagonal cells (HEX), and coefficient of variation (CV). The data collected by the smartphone imaging device was compared to the values from the image created by the microscope. | Data confirm that there is high agreement between ECD, HEX, and CV values when comparing microscope images and imaging device images. | Slit lamp microscope and smartphone. | ECD, HEX, and CV calculated by the Tomey Specular Microscope is used in diagnosing Fuch’s Dystrophy | Mantena et al 2021[^17^](https://www.zotero.org/google-docs/?HNZh8Q) |
| **Gastrointestinal Disorders** | | | | | | |
| Irritable Bowel syndrome (IBS): a disorder that affects the large intestine and the stomach. The symptoms of the Irritable Bowel syndrome includes pain in the belly, gas, diarrhea, and constipation. | A smartphone microphone was placed on the abdomen at a certain angle and location. The audio recorded from the native microphone is used in later analysis. Better microphones will get higher quality audio data. | CNNs were used to detect the presence of bowel sounds in the smartphone recordings. Time-based and frequency-based features were extracted from the higher quality microphone data. This was fed into a logistic regression model to predict IBS and no IBS. | AUC = 0.99 for bowel sound detection from the smartphone. 90% sensitivity and 90% specificity for IBS detection using the higher quality audio. | Smartphone microphone or external microphone. | The ROME III criteria (95% sensitivity and 70% specificity) is the gold standard diagnosis method. It includes symptoms such as abdominal pain, improvement after defecation, change in stool appearance | Kutsumi et al 2023[^18^](https://www.zotero.org/google-docs/?RxR22g), Ford et al 2013[^19^](https://www.zotero.org/google-docs/?udLv7N) |
| Normal and Abnormal Bowel sounds: It is important to characterize the variety of bowel sounds that are created so that abnormal bowel sounds can be identified. | The piezoelectric sensor that connects to a sound recorder was used to collect bowel sounds. It has a form that is similar to a stethoscope. | The bowel sounds recorded from the piezoelectric sensor were categorized and analyzed based on the quantity of each categorical sound and the acoustic features of the sound. An automated bowel sound identification algorithm was used for bowel sound counting. | This showed similar efficacy to electronic stethoscopes for listening to bowel sounds | Piezoelectric sensor and sound recorder | N/A | Du et al 2018[^20^](https://www.zotero.org/google-docs/?kwPqJy) |
| Fecal incontinence: Stool passively passes through the rectum without control from the patient. It can be associated with various gastrointestinal and neurological diseases. | Bowel sounds were detected by an electronic stethoscope for 30 sec two hours after meal and 10 min after defecation. | Collected sounds were processed using a Fourier transform and the resulting frequency domain features were used in a classifier to predict defecation events. | AUC = 0.8 up to 0.974 | Electronic stethoscope | There is no gold standard. The goal of the technology is to help the patient knowing when defecation is going to happen | Marumoto et al 2022[^21^](https://www.zotero.org/google-docs/?iEmbGo) |
| **Neurological Disorders** | | | | | | |
| Alzheimer’s Disease (Eye tracking): progressive neurodegenerative disease that impairs cognition and memory- typically characterized by accumulation of beta amyloid plaques and tau tangles | Eye-tracking equipment can be used to assess the ocular movements of AD patients while conducting specific tasks. Fundamental eye movements and patterns during complex tasks such as visual search and scene exploration were assessed. Other assessments used eye movements during reading tasks to assess AD severity and potentially provide early detection of AD. | Prosaccadic impairments, quantity of incorrect saccades, and smooth pursuit impairments are among some of the many quantifiable impacts of AD on normal eye movements. AD patients also demonstrate reduced amplitude of pupillary changes when compared to healthy controls. In reading tasks, less focused visual explorations and longer fixations were observed. | 91% of participants correctly predicted, 78% sensitivity, and 98% specificity. | Eye tracking sensor | Alzheimer’s Disease diagnoses typically derive from several memory impairment and cognition tests conducted by clinicians. Additionally, structural imaging techniques such as MRI or CT scans are used to assess whether other sources could account for the impairment. | Molitor et al 2017[^22^](https://www.zotero.org/google-docs/?2mpkeh), Fernandez et al 2013[^23^](https://www.zotero.org/google-docs/?K0CQUX) |
| Dementia with Lewy bodies: a neurological disorder characterized by movement and cognition impairment associated with abnormal accumulation of alpha-synuclein the brain. | Color vision impairment can be used to differentiate dementia with lewy bodies from Alzheimer’s disease, as DLB is frequently misdiagnosed. Online 15-hue color vision arrangement tests were administered to patients. | Color vision results were recorded by subtype (protan, deutan, or tritan). Associations between color vision impairment and prodromal phase of AD patients, prodromal phase of DLB patients, or AD patients were established through stepwise multivariate logistic regression analysis. | Significant differences (P<.001) found between DLB, pro-DLB, or pro-AD groups. | Color Vision Tests | Lewy Body Dementia is generally diagnosed from a set of clinical characteristics including fluctuating cognition, visual hallucinations, and Parkinsoninism | Flanigan et al 2018[^24^](https://www.zotero.org/google-docs/?igkPVH) |
| Alzheimer’s Disease (Speech): see above | Patients were asked to tell personal stories, express their feelings, and converse in a friendly way to assess spontaneous speech patterns. The signals were collected using an audio recorder and features were extracted. Spontaneity of these speech patterns were emphasized, as the recording atmospheres were intended to be relaxed and friendly. | Pre-processing to remove laughing, coughing, background noise, and other similar elements were removed from recordings. Bispectrum estimation and bicoherence estimation were used to differentiate between AD patients and healthy controls, comparing these values through T tests and Mann-Whitney U-tests. | Significant differences found between health subjects and AD patients. | Audio recorder | (See above) | Nasrolahzadeh et al 2016[^25^](https://www.zotero.org/google-docs/?45bPYW) |
| Parkinson’s Disease (Speech tremors): a neurodegenerative disease characterized by tremor, muscle rigidity, and fine motor dysfunction | PD patients and normal controls provided voice samples of sustained vowels, numbers, words, and short sentences for the recordings captured by a dedicated microphone. PD patients also provided additional recordings as independent test sets for result validation. | Frequency, pulse, amplitude, voicing, pitch, and harmonicity parameters were extracted from the voice samples, which provided information on subject jitters, number of voice breaks, and other relevant values. Classification with leave-one-subject-out and summarized-leave-one-out methods were conducted on the extracted data. K-nearest neighbor and support vector machines were also used as classification algorithms. | Classification AUC reaches up to 0.951 | Audio recorder | There isn’t a standardized test to diagnose Parkinsons (include the signs/symptoms that doctors look for). The current gold standard is post-death autopsy of a patient's brain. | Ali et al 2019[^26^](https://www.zotero.org/google-docs/?VOzitb) |
| Schizophrenia: a mental disorder characterized by hallucinations, delusions, and disorganized thought | Digital speech recordings were conducted, with schizophrenia patients providing 3 spoken passages. The patients counted out loud from 1 to 40, read an emotionally neutral and simple passage from a children’s book, and counted out loud again from 1 to 40. Other studies only utilize the reading of a children’s book passage, without the counting from 1 to 40 task. | Feature extraction for these studies included separating speech and non-speech sections, counting the number of pauses, mean pause duration, proportion of silence, mean utterance duration, total recording time, total length of pauses, total length of utterances, and relative variation in vocal pitch. Variation of energy/syllable mean vocal pitch, and loudness were also variables considered. | Close associations between psychopathology subscales and acoustic variables, significant differences found. Accuracy ranged from 0.79 to 0.82 depending on the feature set. | Audio recorder | The main method to diagnose Schizophrenia is through the DSM-5 classification. This requires the patient to exhibit two or more symptoms from a list that includes hallucinations, delusions, or disorganized speech for more than a month. | Puschel et al 1998[^27^](https://www.zotero.org/google-docs/?TGKJsr) |
| Alzheimer’s Disease (Writing analysis): see above | The writing tests involve writing short and simple phrases to describe a picture. Linguistic metrics in the cited study use the Cookie Theft Task, a simple and naturalistic language probe from the Boston Aphasia Diagnostic Examination. The data was collected from patients when they were cognitively healthy (before AD onset in some cases). | Metrics such as number of words, number of unique words, misspellings, use of punctuation, uppercasing, and frequencies of repetitions were assessed. A total of 87 linguistic variables were extracted. Neuropsychological tests were included in the predictive models used alongside clinical and demographic variables. | Linguistic variables had classification performance 2.4 times better than non-linguistic variables | Writing implements | (See above) | Eyigoz et al 2020[^28^](https://www.zotero.org/google-docs/?Rs3o9O) |
| Parkinson’s Disease (Drawing Analysis): see above | The drawing test has the patient draw simple figures such as a spiral on a tablet device. The tablet then collects raw data from the drawing including coordinates, pen pressure, and pen angles in relation to the plan of the tablet. | Features are extracted from the raw data including mean pen pressure, maximum velocity, relationship between velocity and curvature, the distribution characteristics of acceleration, etc. These features are then put into a machine learning model to relate the features to the identification of the disorder vs a control. | 98% accuracy for identifying patients with Parkinson’s Disease | Tablet and stylus | (See above) | Chandra et al 2022[^29^](https://www.zotero.org/google-docs/?NrfEGe), Luciano et al 2016[^30^](https://www.zotero.org/google-docs/?PFmGDQ) |
| Attention Deficit Hyperactivity Disorder: a common mental disorder for children, characterized by inability to maintain focus, excessive movement, and impulsivity | Speech recordings were collected during an interview or while the patients completed certain tasks. Recordings were taken using headset microphones or dedicated voice recorders. The content of these samples included free and given speech which assessed voice features and ADHD-symptom severity. | Paralinguistic features were calculated, and random forest-based classifications were applied to the data. Vocalization duration, vocalization frequency, pause frequency, total turn change time (time taken between transitioning speakers), loudness, and other features were assessed. | Classification AUC = 0.76 in one study when controlling for age, sex, and education between patients and healthy controls | Audio recorder | There is no standard way to diagnose ADHD. Diagnostic methods are largely subjective and vary based on clinician. Some questionnaires exist, but typical diagnoses are established by physician judgment. | Ploier et al 2021[^31^](https://www.zotero.org/google-docs/?mzTvGD) |
| Traumatic brain injury: brain injury resulting from sudden trauma (striking of the head, piercing of the skull, jolt to the head, etc.), with concussions being a common example | Recordings for TBI analysis were taken while the patients read a paragraph of text. The recordings were collected using a smartphone microphone. | The recordings were fed through speech recognition pipelines to clean the data for non-verbal features. A deep learning (LSTM) model was used to extract useful information from the audio recordings. | AUC and accuracy were both 0.90 | Audio recorder | TBI’s are typically diagnosed with CAT or CT scans. In addition, patients are interviewed and their responses are judged based on the Glasgow Coma Scale. | Wall et al 2022[^32^](https://www.zotero.org/google-docs/?agU0To) |
| Huntington’s Disease: an inherited neurodegenerative disease that destroys nerve cells in the brain (rate of progression notably depends on the subtype of disease) | Patients are given various oculomotor tasks that assess a subject’s saccades (prosaccade, centrally cued, peripheral conflict). An eye tracker was used to assess the error in their saccades. | Patients with early Huntington’s Disease had significantly increased errors in their saccades in comparison to controls and presymptomatic carriers of the disease. | Given the substantial difference in saccade error, diagnosis and severity tracking are very feasible. | Eye tracker | Normally, a diagnosis of Huntington’s Disease is supported by clinical symptoms and genetic testing. Family history, blood tests, and brain imaging could also be used to assess the risk for a patient. | Hicks et al 2008[^33^](https://www.zotero.org/google-docs/?CHGzyQ) |
| Amyotrophic lateral sclerosis (ALS): a disease targeting the motor neurons of the spinal cord, resulting in progressive weakness and muscular atrophy | No singular test can provide a definitive diagnosis of ALS. The disease is mainly diagnosed after a review of a detailed history of the symptoms observed by a clinician during physical examination plus an analysis of the individual's full medical history, along with a series of tests including electromyogram and nerve conduction studies. However, eye tracking and brain computer interfaces can be used to assist patients with ALS. | Eye movement can be preserved in patients with ALS. As a result, eye tracking technology has been used to allow patients to communicate with their environment. It also allows physicians to assess a patient’s cognitive function. When eye tracking is not possible (low lighting conditions), brain computer interfaces can become useful | N/A | Eye tracker and tablet device | ALS diagnosis typically requires evidence of progressive motor impairment preceded by normal motor function. Additionally, clinicians look at whether motor neuron degeneration has occurred. | Pinto et al 2020[^34^](https://www.zotero.org/google-docs/?wvBOVu) |
| Multiple Sclerosis: disease wherein the immune system targets the nerve fibers and myelin sheathing in the brain and spinal cord leading to vision loss, pain, fatigue, and impaired coordination | Patients were asked to do five different tasks in order to assess their saccadic movement and smooth pursuits. An eye tracker was used to quantify the error and characterize their saccades and smooth pursuits. | Simple measurements including saccadic and anti-saccadic latency were measured using the eye tracker. | Healthy controls had a significantly shorter saccadic latency than patients with Multiple Sclerosis. This indicates eye tracking may be useful in characterizing the disease. | Eye tracker | Initial blood tests combined with structural imaging of the brain through MRI can support an MS diagnosis. The diagnosis of MS has evolved with contributions from the Schumacher criteria, the Poser criteria, and the McDonald criteria. | Yousef et al 2019[^35^](https://www.zotero.org/google-docs/?Ooo0sr) |
| **Geriatrics** | | | | | | |
| Many Different Age-Related Conditions: Stroke, Cardiovascular Disease, Neuropathies and cognitive decline | Grip Strength is a metric that is inversely associated with different poor health outcomes. Clinicians can easily measure grip strength through a hand grip dynamometer. | Cox proportional hazards models controlled for important covariates were used for mortality outcomes. Latent growth models were used to to describe the relationship between grip strength and cognitive scores and predictors of health outcomes | Stroke, Myocardial Infarction, cardiovascular mortality, and non-cardiovascular mortality are significantly associated with grip strength. In addition, key predictors of health outcomes including MAP, gender, marital status, exercise amount, etc. are associated with grip strength. Weakening grip strength was also strongly associated with cognitive decline. | Hand grip dynamometer | N/A | Leong et al 2015[^36^](https://www.zotero.org/google-docs/?G1GjOy), Sternang et al 2014[^37^](https://www.zotero.org/google-docs/?KEjke5), Sternang et al 2016[^38^](https://www.zotero.org/google-docs/?szpb12) |
| **Musculoskeletal Disorders** | | | | | | |
| Rheumatoid Arthritis: an autoimmune disease which typically results in inflammation at joints and painful swelling, leading to bone erosion and joint deformities over time. | Sensor gloves can utilize measurements of hand dexterity, bend, motion, rotation, range of motion, and other kinematic features to diagnose or monitor RA. These gloves typically implement flex sensors at joints to collect this data. Accelerometers also provide hand orientation, rotation of the hand, in addition to the angle, velocity, and acceleration of the finger joints. | Raw data collected from the glove can be related to the presence of arthritis and the severity of arthritis. | Preliminary study without accuracy metrics. | Contact vibration (acceleration) sensors | Radiographic imaging, RF tests, and anti-ccp tests are all used for diagnosis. | Henderson et al 2021[^39^](https://www.zotero.org/google-docs/?nuoQJy) |
| Duchenne’s Muscular Dystrophy: a genetic disorder that results in progressive muscle degeneration and weakness due to abnormalities in the dystrophin protein | Ultrasound small-window entropy imaging to evaluate DMD severity | Envelope amplitude and entropy values extracted from the ultrasound images are used to identify the presence of DMD and the severity of disease. | Diagnostic accuracies up to 90% depending on stage of DMD and the factors included for calculated the AUROCs. | Ultrasound machine (portable) | Biopsies and genetic testing, typically screen for biochemical dysfunction combined with physiological symptoms | Yan et al 2020[^40^](https://www.zotero.org/google-docs/?S77hlk) |
| Carpal Tunnel Syndrome: compression of the median nerve in the hand that can lead to hand/arm weakness and numbness. | The patient is asked to draw a spiral on a tablet. The pressure of the pen and coordinates of the drawing are collected over time. | The jerk of trajectory and pressure are calculated over time. The accuracy of the drawing in relation to the template drawing is also used. This information is used in a machine learning model to detect carpal tunnel syndrome. | 0.81 AUC with when using all of the features. | Tablet and stylus | Nerve conduction studies | Watanabe et al 2021[^41^](https://www.zotero.org/google-docs/?u5IgUE) |
| **Skin Disorders** | | | | | | |
| Skin Cancers, Precancerous Lesions, and other Lesions: Melanoma, Actinic Keratoses, Basal Cell Carcinoma, Dermatofibroma, Melanocytic nevus, Vascular Lesions, Benign keratinocytic lesions. | Dermatoscopic images of various lesions were taken. The images were taken with either normal cameras or a lens attachment to the camera. Simple smartphone cameras can be used to take the images with high enough resolution. | Various deep learning and more classical machine learning models were used to process the images and output the correct type of lesion. | 85% accuracy for the best models. | A smartphone camera | Dermatologists had a 62% accuracy on the same set of images. | Tschandl et al 2019[^42^](https://www.zotero.org/google-docs/?rFUqHi) |
| Other Skin Conditions: 26 of the most common skin conditions including acne, contact dermatitis, cyst, etc. | Images are taken from normal teledermatology cases using Nikon point and shoot cameras or apple iPad minis. | The images are fed into a deep convolutional neural network (CNN). The CNN learns image patterns on the training set and then is tested on a separate testing dataset. The final output is one of the 26 skin conditions in addition to a more specific choice of 419 skin conditions. | 66% overall accuracy using the top prediction only. | Camera or smart device with camera | On the same task, dermatologists had an accuracy of 63% (worse than the CNN) | Liu et al 2020[^43^](https://www.zotero.org/google-docs/?5gqGPO) |
| **Mitochondrial Disorders** | | | | | | |
| Mitochondrial disorders: various complications which cause mitochondria to efficiently produce energy, causing the cells not to work effectively. | Using a sensory mat and camera, researchers were able to analysis velocity, cadence, step length, step time, step time variability, and step width in the recovery condition in patients during the duration of 3 minutes. | When analyzing the previously mentioned variables along with pace (step velocity and step length); rhythm (step time); variability (step length and step time variability); asymmetry (step time asymmetry); and postural stability (step width, step width variability and step length asymmetry), a correlation between most of these variables and if the patient had mitochondrial disorders was found. | Six out of ten parameters showed good or perfect reliability (ICC scores above a .8). | Sensory mat, camera, computer | The standard for diagnosing these mitochondrial disorders is genomic testing. Studies have shown this method as highly accurate. | Galna et al 2014[^44^](https://www.zotero.org/google-docs/?SF6u3g), Koene et al 2018[^45^](https://www.zotero.org/google-docs/?uj4hi2) |
| **Obstetrics Disorders** | | | | | | |
| Fetal Heart Murmurs: unusual heart sounds in the fetus, often indicating abnormalities in the heart's structure or function. These murmurs can be benign or signal serious heart defects, affecting the fetal circulation. | Fetal phonocardiography (fPCG) is employed to detect heart murmurs by capturing the fetal heartbeat sounds using specialized microphones placed on the mother's abdomen. This method is non-invasive and allows for continuous monitoring of fetal heart activity, potentially identifying abnormalities in heart function or structure. | The fPCG signals captured for detecting fetal heart murmurs are analyzed using advanced signal processing and feature extraction techniques. This involves filtering out noise and interference to isolate the heart murmur sounds from normal heartbeats and other background sounds. The refined signals are then fed into classification algorithms such as Support Vector Machines (SVMs) or deep learning models like Convolutional Neural Networks (CNNs), to identify the various types of murmurs. | Accuracy varies from 74.13% - 99% depending on analysis method used in the S1 detection. Most efficient at identifying S1 waveforms. | Phonocardiography device | Ultrasound, with varying accuracies depending on the specific condition and operator experience. | Kahankova et al 2023^46^  Sa-ngasoongsong et al 2012^47^ |
| Intrauterine growth restriction: a condition where a fetus does not grow at the expected rate during pregnancy, leading to a significantly lower birth weight than the normal. | Monitoring of fetal heart rate (FHR) is conducted through cardiotocography. This approach relies on ultrasound for the FHR and a tocodynamometer that measures tension in the abdominal wall. | The FHR signals are analyzed using a combination of linear and nonlinear signal processing techniques. This approach includes the computation of time-domain indices, frequency-domain analyses, and non-linear dynamics, such as entropy measures. The analytical process seeks to identify deviations from normal heart rate patterns that signal the presence of IUGR, enabling early diagnosis and intervention. | There was a significant difference between Intrauterine growth restricted fetuses and normal fetuses based on a few features of the heart rate signal. | Cardiotocography fetal monitor | Ultrasound for IUGR diagnosis, accuracy depends on the timing of diagnosis and measurement techniques. | Signorini et al 2014^48^ |
| Maternal hypoxia: a complication during pregnancy where the mother experiences insufficient oxygen levels, which can lead to reduced oxygen supply to the fetus. | Fetal phonocardiogram signals are obtained from a digital stethoscope and fetal doppler devices. These tools capture the acoustic signals of the fetal heart. | To detect maternal hypoxia through fPCG, two neural network architectures are utilized: a Convolutional Neural Network (CNN) and a network optimized for signal processing challenges, such as a Recurrent Neural Network (RNN) or a Long Short-Term Memory (LSTM) network. These models are trained to identify the S1 waveforms characteristic of a healthy fetal heartbeat, focusing on improving signal-to-noise ratio (SNR) issues present in the raw phonocardiogram signals. | While digital stethoscope gave poor quality data, the fetal doppler yielded much more accurate results for the detection of S1 and S1-S2, with an MAE of .76 and .83 respectively. | Digital stethoscope and fetal doppler | Oxygen saturation monitoring and blood gas analysis, with high accuracy for detecting hypoxia. | Chen et al 2021^49^ |

**Supplemental References**

[1. Yeginer, M. *et al.* Using lung sounds in classification of pulmonary diseases according to respiratory subphases. in *The 26th Annual International Conference of the IEEE Engineering in Medicine and Biology Society* vol. 1 482–485 (2004).](https://www.zotero.org/google-docs/?5qtFWn)

[2. Reichert, S., Gass, R., Brandt, C. & Andrès, E. Analysis of Respiratory Sounds: State of the Art. *Clin. Med. Circ. Respir. Pulm. Med.* **2**, 45–58 (2008).](https://www.zotero.org/google-docs/?5qtFWn)

[3. Gholizadeh, A. *et al.* Toward point-of-care management of chronic respiratory conditions: Electrochemical sensing of nitrite content in exhaled breath condensate using reduced graphene oxide. *Microsyst. Nanoeng.* **3**, 1–8 (2017).](https://www.zotero.org/google-docs/?5qtFWn)

[4. Saglani, S. & Menzie-Gow, A. N. Approaches to Asthma Diagnosis in Children and Adults. *Front. Pediatr.* **7**, (2019).](https://www.zotero.org/google-docs/?5qtFWn)

[5. Imran, A. *et al.* AI4COVID-19: AI enabled preliminary diagnosis for COVID-19 from cough samples via an app. *Inform. Med. Unlocked* **20**, 100378 (2020).](https://www.zotero.org/google-docs/?5qtFWn)

[6. Brown, C. *et al.* Exploring Automatic Diagnosis of COVID-19 from Crowdsourced Respiratory Sound Data. in *Proceedings of the 26th ACM SIGKDD International Conference on Knowledge Discovery & Data Mining* 3474–3484 (2020). doi:10.1145/3394486.3412865.](https://www.zotero.org/google-docs/?5qtFWn)

[7. Mannino, R. G. *et al.* Smartphone app for non-invasive detection of anemia using only patient-sourced photos. *Nat. Commun.* **9**, 4924 (2018).](https://www.zotero.org/google-docs/?5qtFWn)

[8. Ilyas, S., Sher, M., Du, E. & Asghar, W. Smartphone-based Sickle Cell Disease Detection and Monitoring for Point-of-Care Settings. *Biosens. Bioelectron.* **165**, 112417 (2020).](https://www.zotero.org/google-docs/?5qtFWn)

[9. Chorba, J. S. *et al.* Deep Learning Algorithm for Automated Cardiac Murmur Detection via a Digital Stethoscope Platform. *J. Am. Heart Assoc.* **10**, e019905 (2021).](https://www.zotero.org/google-docs/?5qtFWn)

[10. Cao, M. *et al.* Ambulatory Monitoring of Heart Sounds via an Implanted Device Is Superior to Auscultation for Prediction of Heart Failure Events. *J. Card. Fail.* **26**, 151–159 (2020).](https://www.zotero.org/google-docs/?5qtFWn)

[11. Gjoreski, M., Gradišek, A., Budna, B., Gams, M. & Poglajen, G. Machine Learning and End-to-End Deep Learning for the Detection of Chronic Heart Failure From Heart Sounds. *IEEE Access* **8**, 20313–20324 (2020).](https://www.zotero.org/google-docs/?5qtFWn)

[12. Kang, S.-H. *et al.* Cardiac Auscultation Using Smartphones: Pilot Study. *JMIR MHealth UHealth* **6**, e49 (2018).](https://www.zotero.org/google-docs/?5qtFWn)

[13. Dai, L. *et al.* A deep learning system for detecting diabetic retinopathy across the disease spectrum. *Nat. Commun.* **12**, 3242 (2021).](https://www.zotero.org/google-docs/?5qtFWn)

[14. Tan, W. S. *et al.* Individual, clinical and system factors associated with the place of death: A linked national database study. *PLOS ONE* **14**, e0215566 (2019).](https://www.zotero.org/google-docs/?5qtFWn)

[15. Stratton, S. *et al.* Smartphone-based Fundus Photography for Remote Glaucoma Assessment in a Low-Resource Setting. *Invest. Ophthalmol. Vis. Sci.* **62**, 1616 (2021).](https://www.zotero.org/google-docs/?5qtFWn)

[16. Idriss, B. R. *et al.* Smartphone-based Ophthalmic Imaging Compared With Spectral-domain Optical Coherence Tomography Assessment of Vertical Cup-to-disc Ratio Among Adults in Southwestern Uganda. *J. Glaucoma* **30**, e90–e98 (2021).](https://www.zotero.org/google-docs/?5qtFWn)

[17. Mantena, S. *et al.* Low-Cost, Smartphone-Based Specular Imaging and Automated Analysis of the Corneal Endothelium. *Transl. Vis. Sci. Technol.* **10**, 4 (2021).](https://www.zotero.org/google-docs/?5qtFWn)

[18. Kutsumi, Y., Kanegawa, N., Zeida, M., Matsubara, H. & Murayama, N. Automated Bowel Sound and Motility Analysis with CNN Using a Smartphone. *Sensors* **23**, 407 (2022).](https://www.zotero.org/google-docs/?5qtFWn)

[19. Ford, A. C. *et al.* Validation of the Rome III criteria for the diagnosis of irritable bowel syndrome in secondary care. *Gastroenterology* **145**, 1262-1270.e1 (2013).](https://www.zotero.org/google-docs/?5qtFWn)

[20. Du, X., Allwood, G., Webberley, K. M., Osseiran, A. & Marshall, B. J. Bowel Sounds Identification and Migrating Motor Complex Detection with Low-Cost Piezoelectric Acoustic Sensing Device. *Sensors* **18**, 4240 (2018).](https://www.zotero.org/google-docs/?5qtFWn)

[21. Marumoto, S., Kubo, T., Tada, M. & Ikeda, K. Defecation Prediction System Using Bowel Sound. *IPSJ Trans. Bioinforma.* **15**, 17–21 (2022).](https://www.zotero.org/google-docs/?5qtFWn)

[22. Molitor, R. J., Ko, P. C. & Ally, B. A. Eye Movements in Alzheimer’s Disease. *J. Alzheimers Dis. JAD* **44**, 1–12 (2015).](https://www.zotero.org/google-docs/?5qtFWn)

[23. Fernández, G., Laubrock, J., Mandolesi, P., Colombo, O. & Agamennoni, O. Registering eye movements during reading in Alzheimer’s disease: Difficulties in predicting upcoming words. *J. Clin. Exp. Neuropsychol.* **36**, 302–316 (2014).](https://www.zotero.org/google-docs/?5qtFWn)

[24. Flanigan, P. M., Khosravi, M. A., Leverenz, J. B. & Tousi, B. Color Vision Impairment Differentiates Alzheimer Dementia From Dementia With Lewy Bodies. *J. Geriatr. Psychiatry Neurol.* **31**, 97–102 (2018).](https://www.zotero.org/google-docs/?5qtFWn)

[25. Nasrolahzadeh, M., Mohammadpoory, Z. & Haddadnia, J. A novel method for early diagnosis of Alzheimer’s disease based on higher-order spectral estimation of spontaneous speech signals. *Cogn. Neurodyn.* **10**, 495–503 (2016).](https://www.zotero.org/google-docs/?5qtFWn)

[26. Ali, L., Zhu, C., Zhou, M. & Liu, Y. Early diagnosis of Parkinson’s disease from multiple voice recordings by simultaneous sample and feature selection. *Expert Syst. Appl.* **137**, 22–28 (2019).](https://www.zotero.org/google-docs/?5qtFWn)

[27. Püschel, J., Stassen, H. H., Bomben, G., Scharfetter, C. & Hell, D. Speaking behavior and speech sound characteristics in acute schizophrenia. *J. Psychiatr. Res.* **32**, 89–97 (1998).](https://www.zotero.org/google-docs/?5qtFWn)

[28. Eyigoz, E., Mathur, S., Santamaria, M., Cecchi, G. & Naylor, M. Linguistic markers predict onset of Alzheimer’s disease. *EClinicalMedicine* **28**, 100583 (2020).](https://www.zotero.org/google-docs/?5qtFWn)

[29. Chandra, J. *et al.* Screening of Parkinson’s Disease Using Geometric Features Extracted from Spiral Drawings. *Brain Sci.* **11**, 1297 (2021).](https://www.zotero.org/google-docs/?5qtFWn)

[30. Luciano, M. S. *et al.* Digitized Spiral Drawing: A Possible Biomarker for Early Parkinson’s Disease. *PLOS ONE* **11**, e0162799 (2016).](https://www.zotero.org/google-docs/?5qtFWn)

[31. Polier, G. G. von *et al.* Predicting adult Attention Deficit Hyperactivity Disorder (ADHD) using vocal acoustic features. 2021.03.18.21253108 Preprint at https://doi.org/10.1101/2021.03.18.21253108 (2021).](https://www.zotero.org/google-docs/?5qtFWn)

[32. Wall, C. *et al.* A deep learning-based approach to diagnose mild traumatic brain injury using audio classification. *PLOS ONE* **17**, e0274395 (2022).](https://www.zotero.org/google-docs/?5qtFWn)

[33. Hicks, S. L., Robert, M. P. A., Golding, C. V. P., Tabrizi, S. J. & Kennard, C. Chapter 6.9 - Oculomotor deficits indicate the progression of Huntington’s Disease. in *Progress in Brain Research* (eds. Kennard, C. & Leigh, R. J.) vol. 171 555–558 (Elsevier, 2008).](https://www.zotero.org/google-docs/?5qtFWn)

[34. Pinto, S., Quintarelli, S. & Silani, V. New technologies and Amyotrophic Lateral Sclerosis – Which step forward rushed by the COVID-19 pandemic? *J. Neurol. Sci.* **418**, 117081 (2020).](https://www.zotero.org/google-docs/?5qtFWn)

[35. Yousef, A. *et al.* Subclinical Saccadic Eye Movement Dysfunction in Pediatric Multiple Sclerosis. *J. Child Neurol.* **34**, 38–43 (2019).](https://www.zotero.org/google-docs/?5qtFWn)

[36. Leong, D. P. *et al.* Prognostic value of grip strength: findings from the Prospective Urban Rural Epidemiology (PURE) study. *The Lancet* **386**, 266–273 (2015).](https://www.zotero.org/google-docs/?5qtFWn)

[37. Sternäng, O. *et al.* Factors associated with grip strength decline in older adults. *Age Ageing* **44**, 269–274 (2015).](https://www.zotero.org/google-docs/?5qtFWn)

[38. Sternäng, O. *et al.* Grip Strength and Cognitive Abilities: Associations in Old Age. *J. Gerontol. Ser. B* **71**, 841–848 (2016).](https://www.zotero.org/google-docs/?5qtFWn)

[39. Henderson, J., Condell, J., Connolly, J., Kelly, D. & Curran, K. Review of Wearable Sensor-Based Health Monitoring Glove Devices for Rheumatoid Arthritis. *Sensors* **21**, 1576 (2021).](https://www.zotero.org/google-docs/?5qtFWn)

[40. Yan, D. *et al.* Clinical Evaluation of Duchenne Muscular Dystrophy Severity Using Ultrasound Small-Window Entropy Imaging. *Entropy* **22**, 715 (2020).](https://www.zotero.org/google-docs/?5qtFWn)

[41. Watanabe, T. *et al.* The Accuracy of a Screening System for Carpal Tunnel Syndrome Using Hand Drawing. *J. Clin. Med.* **10**, 4437 (2021).](https://www.zotero.org/google-docs/?5qtFWn)

[42. Tschandl, P. *et al.* Comparison of the accuracy of human readers versus machine-learning algorithms for pigmented skin lesion classification: an open, web-based, international, diagnostic study. *Lancet Oncol.* **20**, 938–947 (2019).](https://www.zotero.org/google-docs/?5qtFWn)

[43. Liu, Y. *et al.* A deep learning system for differential diagnosis of skin diseases. *Nat. Med.* **26**, 900–908 (2020).](https://www.zotero.org/google-docs/?5qtFWn)

[44. Galna, B. *et al.* Discrete gait characteristics are associated with m.3243A>G and m.8344A>G variants of mitochondrial disease and its pathological consequences. *J. Neurol.* **261**, 73–82 (2014).](https://www.zotero.org/google-docs/?5qtFWn)

[45. Koene, S. *et al.* Quantification of gait in children with mitochondrial disease. *J. Inherit. Metab. Dis.* **41**, 731–740 (2018).](https://www.zotero.org/google-docs/?5qtFWn)

46. Kahankova, R. *et al.* A Review of Recent Advances and Future Developments in Fetal Phonocardiography. *IEEE Rev Biomed Eng* **16**, 653–671 (2023).

47. Sa-ngasoongsong, A., Kunthong, J., Sarangan, V., Cai, X. & Bukkapatnam, S. T. S. A Low-Cost, Portable, High-Throughput Wireless Sensor System for Phonocardiography Applications. *Sensors (Basel)* **12**, 10851–10870 (2012).

48. Signorini, M. G., Fanelli, A. & Magenes, G. Monitoring fetal heart rate during pregnancy: contributions from advanced signal processing and wearable technology. *Comput Math Methods Med* **2014**, 707581 (2014).

49. Chen, Y. *et al.* Toward Automated Analysis of Fetal Phonocardiograms: Comparing Heartbeat Detection from Fetal Doppler and Digital Stethoscope Signals. *Annu Int Conf IEEE Eng Med Biol Soc* **2021**, 975–979 (2021).
